# Increased carotid intima-media thickness in patients with radiographic axial spondyloarthritis compared to controls and associations with markers of inflammation

**DOI:** 10.1007/s10067-024-06913-8

**Published:** 2024-03-06

**Authors:** Lucy Law, Per Lindqvist, Per Liv, Urban Hellman, Kristina Lejon, Mats Geijer, Stefan Söderberg, Helena Forsblad-d’Elia

**Affiliations:** 1 Department of Public Health and Clinical Medicine, Unit of Medicine, Umeå University, Umeå, Sweden; 2 Department of Surgical and Perioperative Sciences, Clinical Physiology, Umeå University, Umeå, Sweden; 3 Department of Public Health and Clinical Medicine, Section of Sustainable Health, Umeå University, Umeå, Sweden; 4 Department of Clinical Microbiology, Umeå University, Umeå, Sweden; 5 Department of Radiology, Institute of Clinical Sciences, Sahlgrenska Academy, University of Gothenburg, Gothenburg, Sweden; 6 Department of Radiology, Region Västra Götaland, Sahlgrenska University Hospital, Gothenburg, Sweden; 7 Department of Clinical Sciences, Lund University, Lund, Sweden; 8 Department of Rheumatology and Inflammation Research, Institute of Medicine, Sahlgrenska Academy, University of Gothenburg, Gothenburg, Sweden; 9 Clinic of Rheumatology, Sahlgrenska University Hospital, Gothenburg, Sweden

**Keywords:** Cardiovascular disease (CVD), Carotid intima-media thickness (cIMT), Radiographic axial spondyloarthritis (r-axSpA), Ultrasound

## Abstract

**Objective:**

There is an increased risk for cardiovascular disease (CVD) in patients with radiographic axial spondyloarthritis (r-axSpA). In this cross-sectional study, we aimed to, overall and stratified by sex, (i) compare ultrasound derived carotid intima media thickness (cIMT), between patients and controls, and (ii) investigate associations between cIMT, clinical disease activity and inflammation-related laboratory markers in patients with r-axSpA.

**Method:**

In total, 155 patients diagnosed with r-axSpA using the modified New York criteria and 400 controls were included. Bilateral carotid ultrasound, laboratory testing, and questionaries were acquired. Disease-specific assessments were carried out for patients. Linear regression analysis was used to assess associations.

**Results:**

Linear regression analyses showed that patients with r-axSpA had increased mean cIMT compared to controls (mean ± SD, 0.8 ± 0.1 mm vs 0.7± 0.1 mm, respectively, unstandardized *β* (95% CI) -0.076 (-0.10, -0.052), *P* < 0.001) adjusted for smoking status and age. Linear regression analyses for patients with r-axSpA showed that only males presented significant associations between cIMT and inflammation-related laboratory markers, white blood cell (WBC) count (mean ± SD, 6.8 ± 1.6 10^9^/L) and monocytes (0.6 ± 0.2 10^9^/L); WBC count (unstandardized *β* (95% CI) 0.019 (0.0065, 0.031), *P* = 0.003, *R*^2^ = 0.57) and monocytes (0.13 (0.0047, 0.26), *P* = 0.041, *R*^2^ = 0.55), adjusted for age, smoking status, body mass index, hypertension, dyslipidemia, diabetes mellitus, ASDAS-CRP, and treatment with DMARDs and glucocorticoids. No significant association was found between cIMT and clinical disease activity assessed by ASDAS-CRP.

**Conclusion:**

Patients with r-axSpA had significantly increased cIMT compared to controls. In male patients, higher WBC and monocyte count were associated with an increase in cIMT suggesting the role of inflammation in the development of atherosclerosis.

**Table:** 

**Key Points** •*Carotid intima-media thickness was increased in patients with radiographic axial spondyloarthritis compared to controls.* •*White blood cell and monocyte counts were associated with carotid intima-media thickness in male patients with radiographic axial spondyloarthritis.*

**Supplementary Information:**

The online version contains supplementary material available at 10.1007/s10067-024-06913-8.

## Introduction

Radiographic axial spondyloarthritis (r-axSpA), also known as ankylosing spondylitis (AS), is a chronic inflammatory rheumatic disease predominantly affecting the axial skeleton, mainly the sacroiliac joints and spine. r-axSpA often starts in the 3–4th decade of life and is more common in males than females. An increased risk for cardiovascular disease (CVD), including manifestations related to atherosclerosis, has been recognised in r-axSpA patients compared to the general population [1, 2]. Increased carotid intima media thickness (cIMT) is an indicator of subclinical cardiovascular (CV) pathology and considered a precursor to atherosclerosis and a predictor of CV events. This, amongst other changes, leads to augmentation and recruitment of adhesion molecules, and the differentiation of monocytes to macrophages. Macrophages, in turn, secret more proinflammatory cytokines, creating a positive feedback loop which maintains and amplifies the chronic inflammatory state [3, 4]. Over time, the dysfunctional endothelial cells attract substances, such as cholesterol and fibrin, which lead to vessel wall thickening and stiffness, as well as the formation of plaques that can cause vessel stenosis, or embolise resulting in major adverse cardiovascular events (MACE) [5, 6].

Carotid ultrasound is a rapid, reproducible, non-invasive, and safe method to measure cIMT and to screen for atherosclerotic changes, providing additional information to traditional risk factors for CVD [7, 8]. Furthermore, a consensus has been reached regarding the standardisation of the ultrasound procedure used to assess cIMT, the Mannheim Carotid Intima-Media and Plaque Consensus. Thus, there are specific criteria for ultrasound-based cIMT assessment, which ensures consistency in the imaging, measurement, and interpretation of the data [9].

As atherosclerosis is a significant contributor to mortality and morbidity in axSpA, it is important to improve knowledge regarding factors influencing its development in r-axSpA [10]. Additionally, the European League Against Rheumatism (EULAR) stresses the need for proper CVD risk management to ultimately decrease the CVD burden in these patients [10].

The interrelationship between cIMT and r-axSpA has previously been investigated, but the results are conflicting. Thus, more research into cIMT in r-axSpA is warranted [11]. Additionally, determining markers associated with subclinical atherosclerotic disease changes could improve early intervention and management strategies against CVD in patients with r-axSpA [12, 13].

The primary objectives of this cross-sectional study were firstly to conduct a comparative analysis of cIMT using ultrasound imaging in patients diagnosed with r-axSpA in comparison to a control group, overall and stratified by sex; and secondly, to investigate the associations between cIMT measurements and both clinical disease activity and inflammation-related laboratory markers in patients with r-axSpA, overall and stratified by sex.

## Materials and methods

### Patients

Data from 155 patients was obtained from the Backbone study, a study which was designed to investigate disease severity and comorbidities in patients with r-axSpA. This cohort, and the process of inclusion, have previously been described [14]. For patients to be invited to participate, they were (i) diagnosed with r-axSpA according to the modified New York criteria [15], (ii) between 18 and 75 years of age, (iii) had attended the Rheumatology clinic at Umeå University Hospital in northern Sweden (Region Västerbotten) in the last 5 years, (iv) had sufficient knowledge of the Swedish language, and (v) had no other rheumatological disease. All patients underwent an assessment of cIMT with ultrasound. Additionally, patients underwent clinical examination and answered questionnaires regarding lifestyle habits, medication, r-axSpA-related data such as a history of anterior uveitis, peripheral arthritis, and CV-related events such as previous myocardial infarction (MI), surgical myocardial revascularisation or stroke.

Patients having been told by a physician to have hypertension and being on an antihypertensive drug were defined as having hypertension. Patients having been told by a physician to have diabetes mellitus and being on an antidiabetic medication were defined as having diabetes mellitus. Patients currently on lipid lowering medication were classified as having dyslipidaemia. Smoking was categorised as either current (smoking now) or not. The Bath Ankylosing Spondylitis Activity Index (BASDAI), Ankylosing Spondylitis Disease Activity Score with C-reactive protein (ASDAS-CRP), Bath Ankylosing Spondylitis Functional Index (BASFI) and Bath Ankylosing Spondylitis Metrology Index (BASMI) were assessed [16]. Spinal radiographs were performed and scored using the modified stroke ankylosing spondylitis spinal score (mSASSS) by one expert (MG). Blood samples were drawn in the morning after an overnight fast and erythrocyte sedimentation rate (ESR), high-sensitivity (hs) CRP, lipids, and white blood cell (WBC) counts (including lymphocytes, monocytes, basophils, eosinophils, and neutrophils) were analysed by standard laboratory techniques. Additionally, the Discovery U-plex platform (Meso Scale Discovery® (MDS) Rockville, USA) was used to measure Interleukin-6 (IL-6) in plasma according to the manufacturer’s instructions.

### Comparison between r-axSpA patients and controls

As the control group used in this study (described below) had an age range of 53–69 years, patients falling into this age range were selected for comparison with controls. Additionally, the lower age limit was extended by 5 years (i.e., the age range for patients was 48–69 years) to maximize the number of patients used in the comparison, whilst maintaining a fair and accurate statistical assessment.

### Controls

The control group consisted of individuals from the greater Umeå municipality, part of Region Västerbotten, who had previously participated in the Swedish CardioPulmonary bioImaging Study (SCAPIS). Details of the design, participant selection, and purpose of SCAPIS have been published [17]. From the original total number of participants in Umeå (*n* = 2507), 400 individuals who had noted their interest in participating in future studies were asked to take part in this extension study. Details about the inclusion process are found in Supplementary Information (SI) online resource Fig. 1. At the time of original inclusion into SCAPIS, the participants were 50–65 years old, and on inclusion into this extension study participants were 53–69 years old. The controls underwent cIMT assessment with ultrasound and answered questionnaires about lifestyle habits. Controls reported if they were currently taking medication for diabetes mellitus, dyslipidemia, or hypertension.

### Ultrasound examination and analysis

The same expert operator (LL) performed all carotid ultrasound assessments, as well as post-processing analysis and data interpretation for all patient and controls. A General Electric (GE) vivid E9 ultrasound machine (GE Healthcare, Boston Massachusetts) with a GE 9L (2.5-8 MHz) linear probe was used. Participants were asked to lie supine and rotate their head approximately 45 degrees away from the side being assessed. The carotid bulb and bifurcation were included as a reference in all images where possible. For accurate, consistent image analysis and classification cIMT, and the definition between cIMT and plaque, was defined per the Mannheim Carotid Intima-Media and Plaque Consensus [9]. This process was repeated bilaterally with a 3–5 beat cine loop image capture to ensure adequate information for analysis (3 lead electrocardiograph with R wave trigger). All offline imaging analysis was performed using EchoPac (GE Healthcare, Boston Massachusetts, version 204). All imaging was stored in Digital Imaging and COmmunications in Medicine (DICOM) format.

### Reliability testing

Another expert operator (EN) analysed 10 randomly selected participants, blinded to their disease status, using the same imaging analysis and measurement criteria specified above. Results were compared by interobserver reliability testing. The calculated inter-class coefficient of variation was 18.9% (EN) and 20.1% (LL) and the interclass correlation coefficient (ICC) was 0.82 (95% CI 0.34, 0.95).

### Statistical methods

Continuous variables are presented as means and standard deviations (SD), and categorical variables are shown as numbers and percentages (%). An independent *t*-test was used to compare continuous variables and the Chi-square test was used for categorical comparisons. Linear regression analyses were used to assess if disease status (patient or control) was associated with cIMT in unadjusted and adjusted models. Adjustments were made for smoking status, age, and sex and applied to the group overall and stratified by sex. Age was entered into the model as a continuous variable, assuming a linear relationship with cIMT. General linear F-tests, as implemented in the *anova* function of the *rms* package within the statistical software R*,* showed no significant improvement when using restricted cubic splines for modelling age as a non-linear effect compared to assuming linearity; thus, age was assumed to have linear effects throughout this study.

Associations between markers of inflammation and cIMT in patients were assessed using linear regression analyses with cIMT as dependent variable and the markers of inflammation as independent variable, respectively. Three different adjustment models of covariates were used; model 1: unadjusted; model 2: age, sex, smoking status; model 3: age, sex, smoking status, ASDAS-CRP, body mass index (BMI), hypertension, dyslipidemia, and diabetes mellitus. To have a characteristic was coded 1 and to not have a characteristic was coded 0. Female sex was coded 1 and male sex 0. Pharmacological treatment for r-axSpA was dichotomised into current treatment with glucocorticoids, and/or disease modifying anti-rheumatic drugs (DMARDs) (1), or no such treatments (0). A history of CV event(s) was also dichotomised into previous stroke and/or, MI and/or, surgical myocardial revascularisation (1), or no such events or interventions (0).

The inflammatory markers were modelled as continuous variables assuming linear effects. Linearity assumptions were evaluated as previously described. Normality assumptions were verified from visual inspection of histograms and qq-plots of model residuals. Logarithmic transformation was applied to 3 independent variables (hs-CRP, ESR and IL-6) to reduce skewness. *P* < 0.05 was considered statistically significant in all analyses. Statistical analysis was performed using SPSS Statistics package (version 28.0.1.1 (14), IBM, Armonk, NY, USA) and R statistical software package *rms* (version 4.3.1, R Core Team, R Foundation for Statistical Computing, Vienna, Austria).

### Sensitivity testing

Patients with r-axSpA who had reported a previous CV event (*n* = 9) were removed from the analysis, leaving a total of 146 patients. Regression analysis was then performed using the same adjustment models.

## Results

### Characteristics of patients with r-axSpA in the Backbone study

Table 1 shows the descriptive characteristics of the patients in the Backbone cohort overall and stratified by sex. Age, symptom duration, the reported history of CV events, and drug treatment for r-axSpA were similar between the sexes. Females with r-axSpA had lower mean cIMT (0.7 ± 0.1 mm vs 0.8± 0.1 mm, *P* = 0.02) values compared to male patients. BASDAI (4.2 ± 1.8 vs 3.5 ± 1.9, *P* = 0.04) and ESR (16.4 ± 11.5 mm/h vs 12.7 ± 11.8 mm/h, *P* = 0.03) were higher in females compared to males. Male patients showed higher hs-CRP (5.2 ± 6.9 mg/L vs 3.4 ± 3.4 mg/L, *P* = 0.05) and monocyte count (0.6 ± 0.2 10^9^/L vs 0.5 ± 0.210^9^/L, *P* = 0.02) compared to female patients. In supplementary information (SI) Table 1, descriptive characteristics of the 115 patients who were compared with controls, and the 40 patients excluded from comparison with controls are displayed. Compared to the patients excluded from comparison, the 115 patients were significantly older (60.9 ± 7.1 years vs 40.0 ± 6.0 years, *P* < 0.001), had a higher frequency of comorbidities, and higher BASMI and BASFI levels.

**Table 1:** Characteristics of patients with radiographic axial spondyloarthritis in the Backbone study, overall and stratified by sex

	TOTAL (*n* = 155)	Male (*n* = 107)	Female (*n* = 48)	*P* values
Age, years	55.5 ± 11.4	54.5 ± 11.7	57.7 ± 10.6	0.11
BMI, kg/m^2^	27.9 ± 5.3	28.2 ± 5.6	27.1 ± 4.6	0.21
Smoking status				
Current smoker	8 (5.2) °	5 (4.7)	3 (6.3)	0.68
r-axSpA related variables				
Duration of symptoms, years	31.8 ± 11.9	31.1 ± 11.8	33.5 ± 12.1	0.25
HLA B27-positive	153 (98.1)	105 (98.1)	47 (97.9)	0.93
History of anterior uveitis	80 (51.6)	54 (50.5)	26 (54.2)	0.67
History of peripheral arthritis	83 (53.5)	54 (50.5)	29 (60.4)	0.25
BASDAI	3.7 ± 1.9	3.5 ± 1.9	4.2 ± 1.8	**0.04**
ASDAS-CRP	1.8 ± 0.7	1.8 ± 0.7	1.9 ± 0.7	0.64
BASFI	3.0 ± 2.0	2.8 ± 2.0	3.3 ± 2.1	0.15
BASMI	4.1 ± 1.6	4.2 ± 1.6	4.0 ± 1.4	0.48
NSAID, daily use	76 (49.0)	54 (50.5)	22 (45.8)	0.18
csDMARD	19 (12.3) °	14 (13.1)	5 (10.4) °	0.31
bDMARD	25 (16.3)	18 (16.8) °	7 (14.6) °	0.80
csDMARD and/or bDMARD	38 (24.5)	25 (23.4)	13 (27.1)	0.62
Glucocorticosteroids	36 (23.2)	24 (22.4)	12 (25.0) °	0.68
r-axSpA drug treatment^#^	58 (37.4)	40 (37.4)	18 (37.5)	0.99
mSASSS	18.0 ± 20.7 ^	21.6 ± 21.5^	10.0 ± 16.6	**0.001**
Comorbidity and CV-related variables				
SBP, mmHg	136.1 ± 17.6	136.9 ± 16.4	134.4 ± 20.0	0.41
DBP, mmHg	76.6 ± 9.9	77.1 ± 10.2	75.5 ± 9.2	0.36
Hypertension	69 (44.5)	46 (43.0)	23 (48.0)	0.57
Previous myocardial infarction	6 (3.9)	6 (5.6)	0 (0.0)	0.09
Surgical myocardial revascularisation	5 (3.2)	4 (3.7)	1 (2.1)	0.59
Previous stroke	2 (1.3)	1 (0.9)	1 (2.1)	0.56
Previous CV event^§^	9 (5.8)	7 (6.5)	2 (4.2)	0.56
Diabetes mellitus	11 (7.1)	10 (9.3)	1 (2.1)	0.10
Dyslipidemia	22 (14.2)	17 (15.9)	5 (10.4)	0.38
Left cIMT, mm	0.8 ± 0.2°	0.8 ± 0.2°	0.7 ± 0.2	**0.01**
Right cIMT, mm	0.7 ± 0.2	0.8 ± 0.1	0.7 ± 0.2	**0.05**
Mean cIMT, mm	0.8 ± 0.1°	0.8 ± 0.1°	0.7 ± 0.1	**0.02**
Markers of inflammation				
hs-CRP, mg/L	4.6 ± 6.1	5.2 ± 6.9	3.4 ± 3.4	**0.05**
ESR, mm/h	13.8 ± 11.8	12.7 ± 11.8	16.4 ± 11.5	**0.03**
IL-6, pg/mL	2.4 ± 7.9	3.0 ± 9.5	1.1 ± 0.7	0.09
WBCs, 10^9^/L	6.9 ± 1.8	6.8 ± 1.6	7.1 ± 2.0	0.17
Monocytes, 10^9^/L	0.6 ± 0.2	0.6 ± 0.2	0.5 ± 0.2	**0.02**
Lymphocytes, 10^9^/L	1.9 ± 0.6	1.9 ± 0.6	2.0 ± 0.7	0.15
Basophils, 10^9^/L	0.1 ± 0.0	0.5 ± 0.2	0.1 ± 0.0	0.49
Eosinophils, 10^9^/L	0.2 ± 0.1	0.2 ± 1.4	0.2 ± 0.1	0.18
Neutrophils, 10^9^/L	4.2 ± 1.5	4.1 ± 1.4	4.4 ± 1.6	0.18

Values are mean ± SD or n (%)

*r-axSpA* radiographic axial spondyloarthritis, *BMI* body mass index, HLA B-27 human leukocyte antigen B-27, *BASDAI* bath ankylosing disease activity index, *ASDAS-CRP* ankylosing spondylitis disease activity score with c-reactive protein, *BASFI* bath ankylosing spondylitis functional index, *BASMI* bath ankylosing spondylitis metrology index, *NSAID* nonsteroidal anti-inflammatory drug, *csDMARD* conventional synthetic disease-modifying antirheumatic drug, *bDMARD* biological disease-modifying antirheumatic drugs, *SBP* systolic blood pressure, *DBP* diastolic blood pressure, *CV* cardiovascular, *cIMT* carotid intima media thickness, *hs-CRP* high sensitivity C-reactive protein, *ESR* erythrocyte sedimentation rate, *IL-6* interlukin-6, *WBCs* White blood cells

^§^CV event variable is combined incidence of myocardial infarction, stroke and surgical myocardial revascularisation variables

^#^r-axSpA drug treatment is a combined variable including treatment with DMARDs and/or glucocorticosteroids

°1 value missing

^2 values missing

### Comparisons of characteristics between patient with r-axSpA and controls

In Table 2, comparisons of descriptive data between patients with r-axSpA and controls is shown. The sex distribution was significantly different between patients and controls with fewer females in the r-axSpA group (33.0%) compared with controls (51.0%), whereas no difference in mean age was found. Significantly more patients were treated for hypertension than controls (53.0% vs 40.8%, *P* = 0.02). Patients had significantly increased left, right and overall mean cIMT (0.8 ± 0.1 mm vs 0.7± 0.1 mm, *P* < 0.001), as well as weighed significantly more than controls (83.4 ± 19.6 kg vs 80.2 ± 17.1 kg, *P* < 0.001).

**Table 2:** Comparisons of descriptive data between patients with radiographic axial spondyloarthritis and controls

	Patients (*n* = 115)	Controls (*n* = 400)	*P*-value
Sex			
Male	77 (67.0)	196 (49.0)	** < 0.001**
Female	38 (33.0)	204 (51.0)	** < 0.001**
General characteristics			
Age, years	60.9 ± 7.1	62.3 ± 4.2	0.30
Height, cm	171.4 ± 9.4	171.5 ± 9.6	0.90
Weight, kg	83.4 ± 19.6	80.2 ± 17.1	** < 0.001**
BMI, m^2^	28.3 ± 5.5	27.2 ± 4.8	0.31
Smoking status^#^			
Current	8 (7.0) *	21 (5.3) #	0.51
Comorbidity and cardiovascular related variables			
SBP, mmHg	140.0 ± 17.9	127.0 ± 15.0	** < 0.001**
DBP, mmHg	77.0 ± 9.7	79.0 ± 7.5	** < 0.001**
Hypertension	61 (53.0)	163 (40.8)	**0.02**
Diabetes mellitus	11 (9.6)	30 (7.5) *	0.48
Dyslipidemia	21 (18.3)	99 (24.8)	0.15
Left cIMT, mm	0.8 ± 0.2*	0.7 ± 0.1^°^	** < 0.001**
Right cIMT, mm	0.8 ± 0.1	0.7 ± 0.1^“^	** < 0.001**
Mean cIMT, mm	0.8 ± 0.1*	0.7 ± 0.1^€^	** < 0.001**

Values are mean ± SD or numbers of patients and percent (%)

*BMI* body mass index, *S/DBP* systolic/diastolic blood pressure, *cIMT* carotid intima media thickness

^*^1 value missing

^2 values missing

^#^3 values missing

^°^5 values missing

^“^11 values missing

^€^16 values missing

### Linear regression models with cIMT as the dependent variable and disease status as an independent factor

Table 3 shows the results of linear regression analyses exploring factors associated with cIMT in patients with r-axSpA and controls, overall and stratified by sex. For all participants overall, and stratified by sex, all models showed that significantly higher left, right and overall mean cIMT values were associated with r-axSpA.

**Table 3:** Linear regression models showing association between left, right, and mean cIMT with disease status, overall and stratified by sex

		Left cIMT, mm		Right cIMT, mm		Mean cIMT, mm	
		B, unstandardized (CI 95%)	*P*	B, unstandardized (CI 95%)	*P*	B, unstandardized (CI 95%)	*P*
All participants	Model 1	-0.073 (-0.10, -0.43)^°^ °°	** < 0.001**	-0.070 (-0.098, -0.043)”	** < 0.001**	-0.072 (-0.097, -0.047)^€^	** < 0.001**
	Model 2	-0.077 (-0.11, -0.047)^°^ °°	** < 0.001**	-0.076 (-0.10, -0.048)”	** < 0.001**	-0.076 (-0.10, -0.052) ^€^	** < 0.001**
	Model 3	-0.067 (-0.096, -0.038)°	** < 0.001**	-0.066 (-0.093, -0.039)”	** < 0.001**	-0.066 (-0.091, -0.042)^€^	** < 0.001**
Males	Model 1	-0.066 (-0.11, -0.028)^%^ °°	** < 0.001**	-0.061 (-0.096, -0.026)^+^	** < 0.001**	-0.064 (-0.096, -0.031)^°^ °°	** < 0.001**
	Model 2	-0.072 (-0.11, -0.033)^%^ °°	** < 0.001**	-0.069 (-0.10, -0.034)^+^	** < 0.001**	-0.070 (-0.10, -0.038)^°^ °°	** < 0.001**
Females	Model 1	-0.056 (-0.10, -0.011)^+^	**0.02**	-0.061 (-0.11, -0.016)^§^	**0.01**	-0.060 (-0.098, -0.021)^*^	**0.002**
	Model 2	-0.056 (-0.10, -0.011)^+^	**0.02**	-0.061 (-0.11, -0.017)^§^	**0.01**	-0.060 (-0.098, -0.022) ^*^	**0.002**

*cIMT* carotid intima media thickness

^%^1 control value missing

°°1 patient value missing

^+^4 control values missing

^°^5 control values missing

^§^7 control values missing

^*^8 control values missing

^“^11 control values missing

^€^16 control values missing

Adjustment models:

1. Disease status

2. Adjusted for disease status, smoking status and age (assumed linear)

3. Adjusted for disease status, smoking status, age and sex

### Linear regression analyses exploring associations between markers of inflammation and disease activity with mean cIMT in the Backbone cohort

**Fig. 1:**
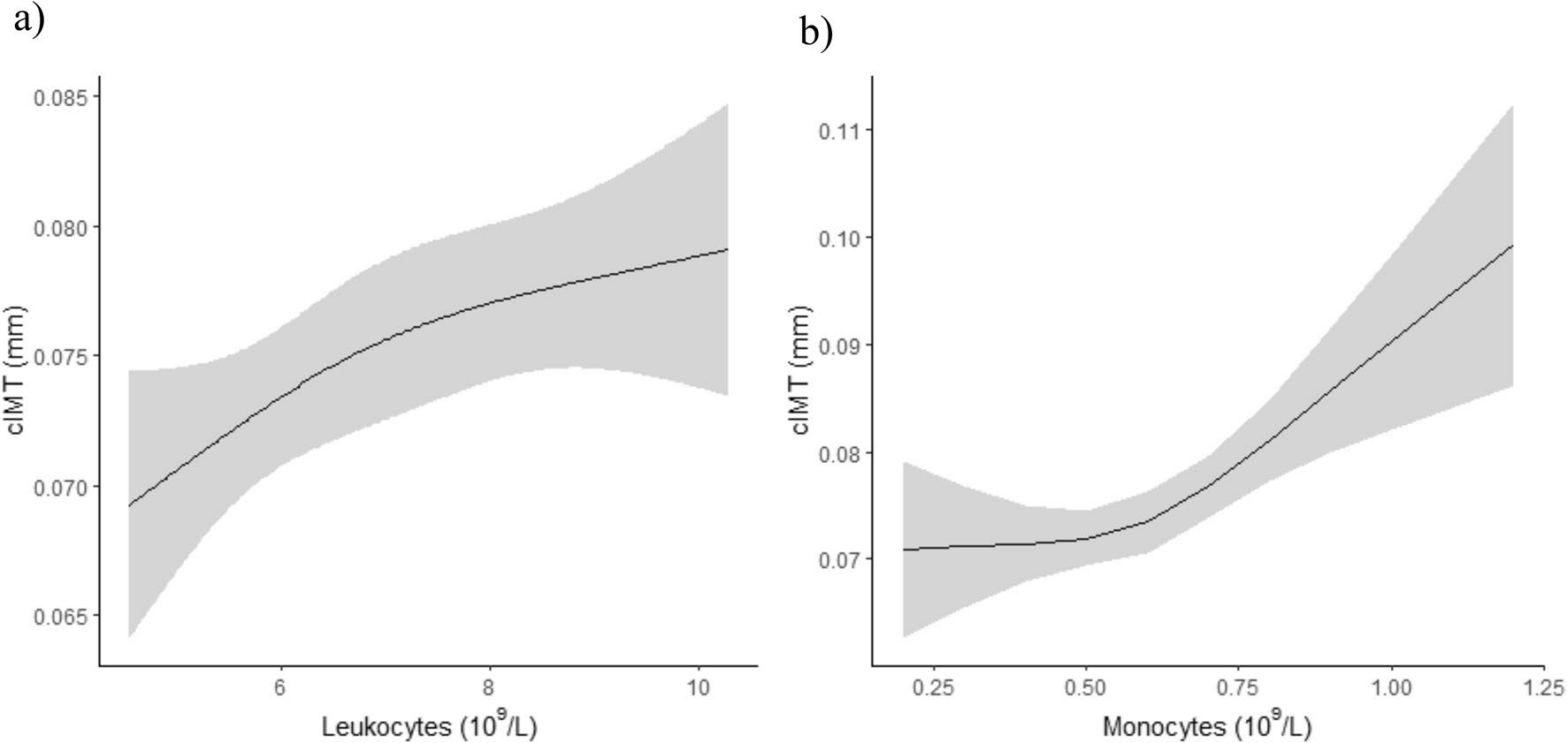
Graphical presentation of spline exploration regarding the relationship between **a**) WBC (leukocytes) and **b**) monocytes with mean cIMT, respectively, in patients with radiographic axial spondyloarthritis in the Backbone study. cIMT: carotid intima media thickness, WBCs: white blood cells

Regression analysis showed no significant associations between mean cIMT and hs-CRP, ESR, IL-6, and ASDAS-CRP in either the unadjusted or adjusted models. In contrast, significant associations were found between the WBC count in all models. For the WBC count, adjustment models 2 (unstandardized *β* (95% CI) 0.017, (0.0073, 0.027) *P* < 0.001, *R*^2^ 0.44; age, sex, and smoking status) and 3 (unstandardized *β* (95% CI) 0.018, (0.0076, 0.028), *P* < 0.001, *R*^2^ 0.48; age, sex, smoking status, ASDAS-CRP, BMI, hypertensive disease, hyperlipidemia medication, diabetic and r-axSpA drug treatment) showed the most significant result. Additionally, a significant association was seen between monocyte count and cIMT in model 1 (unstandardized *β* (95% CI) 0.25, (0.12, 0.38), *P* < 0.001, *R*^2(unadjusted)^ 0.085) and model 2 (unstandardized *β* (95% CI) 0.12, (0.010, 0.23), *P* = 0.033, *R*^2^ 0.41). The results for all models are displayed in Table 4. Figure 1 graphically illustrates spline explorations into the relationship between cIMT with WBC and monocyte counts.

**Table 4:** Linear regression models exploring the relationship between biomarkers of inflammation, disease activity and mean cIMT in patients with radiographic axial spondyloarthritis

		Mean cIMT, mm		
		Regression coefficient, β unstandardized (CI95%)	R^2^ adjusted	*P* value
^§^ hs-CRP, mg/L	Model 1	0.010 (-0.039, 0.058)	0.001^	0.70
	Model 2	-0.0048 (-0.043, 0.034)	0.39	0.81
	Model 3 *	-0.014 (-0.052, 0.024)	0.44	0.47
^§^ ESR, mm/h	Model 1	0.022 (-0.034, 0.077)	0.004^	0.45
	Model 2	-0.0082 (-0.053, 0.037)	0.39	0.72
	Model 3	0.00061 (-0.048, 0.049)	0.44	0.98
^§^ IL-6, pg/mL	Model 1	0.018 (-0.0064, 0.042)	0.014^	0.15
	Model 2	0.0017 (-0.018, 0.021)	0.39	0.86
	Model 3	-0.0028 (-0.023, 0.017)	0.44	0.79
WBCs, 10^9^/L	Model 1	0.017 (0.0039, 0.030)	0.042^	**0.011**
	Model 2	0.017 (0.0073, 0.027)	0.44	** < 0.001**
	Model 3	0.018 (0.0076, 0.028)	0.48	** < 0.001**
Monocytes, 10^9^/L	Model 1	0.25 (0.12, 0.38)	0.085^	** < 0.001**
	Model 2	0.12 (0.010, 0.23)	0.41	**0.033**
	Model 3	0.10 (-0.013, 0.21)	0.45	0.082
ASDAS-CRP	Model 1	-0.0013 (-0.034, 0.032)	0.00^	0.94
	Model 2	-0.0071 (-0.034, 0.020)	0.39	0.56
	Model 3	-0.020 (-0.046, 0.0068)	0.44	0.15

*cIMT *carotid intima media thickness, *CI 95% *95% confidence interval, *hs-CRP *high sensitivity C-reactive protein, *ESR *erythrocyte sedimentation rate, *IL-6 *Interlukin 6, *WBCs *white blood cells, *BMI *body mass index, *ASDAS *ankylosing spondylitis disease activity score, *DMARDs *disease modifying anti-rheumatic drugs, *CV *cardiovascular, *MI *myocardial infarction

^R^2^ Values are unadjusted

^*^ASDAS-CRP was not used in these adjustment models due to co-linearity with hs-CRP

^**§**^Independent variables were log adjusted

Adjustment models

1. Unadjusted

2. Age, sex, smoking status

3. Age, sex, smoking status, ASDAS-CRP, BMI, hypertension, dyslipidemia, diabetes mellitus, r-axSpA drug treatment (DMARDs and/or glucocorticoids)

As a sensitivity analysis, the nine patients with at least one previous CV event or intervention, were excluded from the analyses (online resource SI Table 2). The results were similar to those of the whole Backbone cohort presented above, with the addition of a significant association for model 3 for monocyte count.

Table 5 shows further regression analysis investigations of the above significant associations stratified by sex. For males, all models showed significant associations. For females, no significant associations between mean cIMT and WBC and monocyte counts were seen in any of the models.

**Table 5:** Linear regression models exploring the relationship between WBCs and monocytes, disease activity and mean cIMT in patients with radiographic axial spondyloarthritis stratified by sex

		Mean cIMT, mm					
		Males			Females		
		Regression coefficient, β unstandardized (CI95%)	R^2^ adjusted	*P* value	Regression coefficient, β unstandardized (CI95%)	R^2^ adjusted	*P* value
WBCs, 10^9^/L	Model 1	0.020 (0.0035, 0.036)	0.053^	**0.018**	0.015 (-0.0059, 0.035)	0.043^	0.16
	Model 2	0.017 (0.0042, 0.029)	0.46	**0.009**	0.018 (-0.00059, 0.037)	0.30	0.057
	Model 3	0.019 (0.0065, 0.031)	0.57	**0.003**	0.013 (-0.0047, 0.031)	0.38	0.14
Monocytes, 10^9^/L	Model 1	0.26 (0.10, 0.42)	0.094^	**0.001**	0.16 (-0.085, 0.41)	0.037^	0.19
	Model 2	0.13 (0.00032, 0.26)	0.44	**0.049**	0.091 (-0.13, 0.31)	0.25	0.42
	Model 3	0.13 (0.0053, 0.26)	0.55	**0.041**	-0.074 (-0.34, 0.19)	0.35	0.58

*cIMT* carotid intima media thickness, CI 95%: 95% confidence interval, *WBCs* white blood cells, *BMI* body mass index, *ASDAS-CRP* ankylosing spondylitis disease activity score with C reactive Protein, *DMARDs* disease modifying anti-rheumatic drugs, *CV* cardiovascular, MI: myocardial infarction

^R^2^ Values are unadjusted

Adjustment models

1. Unadjusted

2. Age, smoking status

3. Age, smoking status, ASDAS-CRP, BMI, hypertension, dyslipidemia, diabetes mellitus, r-axSpA drug treatment (DMARDs and glucocorticoids)

## Discussion

In this study, we assessed cIMT by ultrasound as an indirect method of evaluating subclinical atherosclerotic changes. We demonstrated that patients with r-axSpA had increased mean and bilateral cIMT, compared to controls, overall and stratified by sex, and that male r-axSpA patients had the most significant results in all models. Furthermore, we found significant associations between cIMT and laboratory markers of inflammation (WBC count and monocyte count) in male but not female patients.

Our cIMT results, comparing patients with r-axSpA with controls, agree with findings presented previously [11, 18–21] but conflict with others [22–25]. However, in a recent systematic review and meta-analysis by Yuan et al. [11], the authors concluded that the majority of current literature on this topic finds that cIMT is significantly increased in patients with r-axSpA compared to controls. The heterogeneity of previous results could be explained by the small sample sizes, varying ethnic backgrounds, and inconsistent data collection and analysis methods. In another systematic review and meta-analysis by Bai et al. [25], the authors review three methods for assessment of subclinical atherosclerosis, including ultrasound derived cIMT, and their relationship to cIMT in patients with r-axSpA. They reported an increase in pulse wave velocity and cIMT, as well as decrease in flow-mediated dilation, which further implies accelerated subclinical atherosclerosis in patients with r-axSpA. Consequently, we support the view of González Mazón et al. [8] who suggest that cIMT in patients with r-axSpA should be taken into consideration when evaluating CVD risk profile, which also concurs with the statement by EULAR [10].

Our regression analyses, that included patients as well as controls, revealed that disease status was associated with increased cIMT, even after adjustment for age and smoking status. Upon stratification by sex, disease status was seen to be associated with increased cIMT in both males and females. Furthermore, upon adjustment of the model for smoking status and age, these associations remained, however were somewhat stronger for males compared to females. This finding is in line with previous studies which have shown males with r-axSpA to be more affected by CVD comorbidities than females [14, 26, 27]. Studies suggest that differences in genetic, immunological, and hormonal factors between males and females might contribute to the increased incidence of CVD in male patients with r-axSpA [28, 29]. To the best of our knowledge, our study is the first to compare cIMT between patients with r-axSpA and controls stratified by sex. Our finding indicates that there might be a sex difference in subclinical atherosclerotic development in r-axSpA patients, with male patients being more affected in the specific age bracket investigated. However, further studies are needed to validate these findings.

Moreover, regression analyses were carried out to evaluate associations between laboratory markers of inflammation and disease activity with cIMT in the Backbone cohort. Analyses revealed significant associations between WBC count and cIMT in all three models, and monocyte count in two models. Additionally, we carried out further regression analyses of the significant findings stratified by sex and found that the significant results only remained for male patients. The variables age, sex, smoking status, ASDAS-CRP, BMI, hypertension, dyslipidemia, diabetes mellitus, r-axSpA drug treatment were used in the adjusted models. Interestingly, despite various adjustment models, regression analyses did not show any significant associations between cIMT and hs-CRP, ESR, or IL-6; in line with the findings of several studies [18, 19, 30–32]. The lack of association may be explained by these biomarkers being largely indicators of acute inflammation that may vary considerably over time and may not be representative of chronic inflammation [21]. Likewise, we did not find a significant association between the level of disease activity measured by ASDAS-CRP and cIMT, which also agreed with previous studies [23, 33]. This again may be because r-axSpA disease activity scores incorporate acute phase reactant values (hs-CRP or ESR) and mainly assess the recent history of disease activity, not necessarily chronic disease activity. Few studies have considered markers of chronic or accumulated inflammation in relation to cIMT in r-axSpA patients [21]; thus, little is known regarding this topic.

The significant associations found between other markers of inflammation and cIMT in the overall group, and stratified by sex are, to our knowledge, the first of their kind. These findings are consistent with previously published literature which states that males with r-axSpA are more prone to CVD related co-morbidities [26, 27]. This result further supports our belief that different factors may affect the development of atherosclerosis in males and females with r-axSpA, however, further research is required to fully explore and understand these factors.

WBCs are immune cells consisting of various subspecialised cell types including basophils, neutrophils, monocytes, eosinophils, and lymphocytes which are important for the body’s immune response. An elevated leukocyte count is thus a broad indicator of systemic inflammation, infection, or disease [34, 35]. It has been established that a high WBC count is associated with, or is a predictor of, various pathologies including CVD [35–37]. Specifically, studies have found that an elevated WBC count is associated with a decrease in endothelial reactivity [38], an early sign of endothelial dysfunction, and have also been associated with increased cIMT [39, 40]. Our study is, to the best of our knowledge, the first to show the association between WBC count and cIMT in r-axSpA patients. We acknowledge that little is known about the intricacies of overall WBC count in r-axSpA and its associations with disease severity and co-morbidities. Longitudinal studies on the role of WBCs as a possible predictor for cIMT development are thus warranted.

Furthermore, we found significant associations between monocyte count and cIMT in male, but not female, patients in the Backbone cohort. Monocytes account for 2–8% of WBCs. They are attracted to damaged or diseased cells, such as dysfunctional endothelial cells, where they differentiate into macrophages. In addition to the phagocytosis of damaged cells, macrophages secret proinflammatory cytokines, such as IL-6, helping to establish a positive feedback loop and consequently a chronic inflammatory response. [41, 42]. Previously, Surdacki et al. found that there were enhanced monocyte-endothelial interactions due to dysregulation of the immune system in r-axSpA patients, and that this was associated with increased risk of CVD in patients with r-axSpA as interactions between monocytes and the endothelium are a known precursor to the development of clinically significant atherosclerosis [43]. Specifically designed studies are required to further investigate our finding about the association between monocyte count and cIMT, which, to our knowledge, has not been presented before in patients with r-axSpA.

There are several limitations to acknowledge: (i) our study is not longitudinal, thus we are unable to comment on causality; (ii) the findings of this study are based primarily on white individuals living in a specific geographical location, thus, the observations might not be generalisable to the wider group of patients with r-axSpA; (iii) the female r-axSpA group was smaller than the male group, which may have resulted in lower statistical power for analyses of the female group; and (iv) details regarding treatment with beta-blockers or angiotensin-converting enzyme inhibitor medication for conditions other than hypertension in control subjects were unknown. Additionally, cIMT associations were investigated for multiple biomarkers. Consequently, we chose not to control the familywise error rate to account for multiplicity as this leads to a decrease in statistical power. However, this may lead to an increase in risk of type 1-errors; thus, caution in interpretation of findings should be taken.

Despite the limitations, there are several strengths. First, to the best of our knowledge, our study is one of the largest cross-sectional studies with region-matched controls investigating factors associated with increased cIMT in r-axSpA patients, overall and stratified by sex. Secondly, our study has been performed using validated methods of data collection and analysis, and all ultrasound data was gathered and processed by a single expert operator (LL), reducing intra-operator variability. Thirdly, *R*^2^ values for regression analysis assessing the associations between leukocyte count and mean cIMT showed moderately high to high values when assessing the entire Backbone cohort and in the sensitivity analysis. Upon stratification by sex, moderately high *R*^2^ values for some of the models were noted for both leukocyte and monocyte counts in male patients.

## Conclusion

In this study, patients with r-axSpA had thicker ultrasound-derived cIMT when compared to controls, overall and stratified by sex. Furthermore, significant associations were observed between cIMT and WBC count, as well as for monocyte count, in male but not female patients from the Backbone cohort. This study uncovers an accessible methodology that combines routine laboratory blood analysis with standard ultrasound-derived cIMT measurements to identify r-axSpA patients at a potentially higher risk of atherosclerotic-related complications.

### Supplementary Information

Below is the link to the electronic supplementary material.

Supplementary file1 (DOCX 54 KB)

## Data Availability

The data sets generated and/or analyzed during the current study are not publicly available due to the General Data Protection Regulation (GDPR), but a limited data set that supports the main analyses is available on reasonable request.
