# Supplementary material for: Increased carotid intima-media thickness in patients with radiographic axial spondyloarthritis compared to controls and associations with markers of inflammation
Source: Clin Rheumatol. 2024 Mar 6;43(5):1559–70. doi: 10.1007/s10067-024-06913-8 (PMC11018678; doi:10.1007/s10067-024-06913-8)
Supplement: Supplementary file 1 — Supplementary file1 (DOCX 54 KB) [file 10067_2024_6913_MOESM1_ESM.docx]

**SUPPLEMENTARY INFORMATION (SI)**

**Title**

Increased carotid intima-media thickness in patients with radiographic axial spondyloarthritis compared to controls and associations with markers of inflammation

**Journal name:**

Clinical Rheumatology

**Authors**

Lucy Law, B.Sc ^1^, Per Lindqvist, B.Sc. Ph.D.^2,^, Per Liv, Ph.D ^3^, Urban Hellman, Ph.D^1^, Kristina Lejon^4^, M.D, Mats Geijer^5,6,7^, Stefan Söderberg, M.D., Ph.D. ^1^, Helena Forsblad-d’Elia, M.D, Ph.D.^1,8,9^

**Author affiliations**

^1^ Department of Public Health and Clinical Medicine, Unit of Medicine, Umeå University, Umeå, Sweden

^2^ Department of Surgical and Perioperative Sciences, Clinical Physiology, Umeå University, Umeå, Sweden

^3^ Department of Public Health and Clinical Medicine, Section of Sustainable Health, Umeå University, Umeå, Sweden

^4^Department of Clinical Microbiology, Faculty of Medicine, Umeå University, Umeå, Sweden

^5^ Department of Radiology, Institute of Clinical Science, Sahlgrenska Academy, University of Gothenburg, Gothenburg, Sweden, ^6^Region Västra Götaland, Sahlgrenska University Hospital, Department of Radiology, Gothenburg, Sweden

^7^ Department of Clinical Science, Lund University, Lund, Sweden

^8^ Department of Rheumatology and Inflammation Research, Institute of Medicine, Sahlgrenska Academy, University of Gothenburg, Gothenburg,  ^9^ Clinic of Rheumatology, Sahlgrenska University Hospital, Gothenburg, Sweden

**Corresponding author**

Lucy Law

E-mail: lucy.law@umu.se

Phone number: +46 730 494459 (SE), +61 473 131 547 (AU)

**Fig. 1.** Flowchart showing the inclusion process of participants into the Swedish CArdioPulmonary bioImaging Study (SCAPIS) extension study.

2507 individuals from the greater Umeå municipality (Region Västerbotten) were included in the SCAPIS study, 2012-2015

Planning of ultrasound imaging follow up study in 2020-2021

“STEP 1” 400 evenly sex distributed SCAPIS participants, who had indicated their willingness to participate in future studies, were randomly selected from the original SCAPIS cohort

80 invited individuals:

- Did not reply to invitation (n=27)
- Agreed but never showed up for assessment (n=4)
- Declined to participate or were unable to participate due to e.g.: work commitments (n=19)
- No longer living in the region (n=7)
- Deceased (n=1)
- Were too sick to attend (n=2)
- Had no valid contact details (n=20)

“STEP 2” Additional individuals were contacted and invited to participate

In total, 400 participants attended clinical assessment, answered questionnaries, and underwent ultrasound assessment

Process repeated until desired number of participants (400) was achieved

**Table 1.** Characteristics and comparisons of patients with radiographic axial spondyloarthritis included and excluded from comparison with controls.

|  | **r-axSpA patients compared with controls** n= 115 | **r-axSpA patients excluded from comparison**  n= 40 | **P-value** |
| --- | --- | --- | --- |
| Male | 77 (67.0) | 30 (75.0) | - |
| Female | 38 (33.0) | 10 (25.0) | - |
| Age, years | 60.9 ± 7.1 | 40.0 ± 6.0 | **<0.001** |
| BMI, kg/m^2^ | 28.3 ± 5.5 | 26.7 ± 4.8 | 0.10 |
| **Smoking status** |  |  |  |
| Current | 8 (7.0) | 0 (0.0) | 0.09 |
| **r-axSpA related variables** |  |  |  |
| Duration of symptoms, years | 36.4 ± 9.4 | 17.5 ± 8.3 | **<0.001** |
| HLA B27-positive | 114 (99.0) | 38 (95.0) | 0.10 |
| History of anterior uveitis | 63 (54.8) | 23 (57.5) | 0.18 |
| History of peripheral arthritis | 65 (56.5) | 18 (45.0) | 0.21 |
| BASDAI | 4.0 ± 1.9 | 3.1 ± 1.6 | **0.01** |
| ASDAS-CRP | 1.9 ± 0.7 | 1.7 ± 0.6 | 0.10 |
| BASFI | 3.3 ± 2.1 | 1.9 ± 1.6 | **<0.001** |
| BASMI | 4.6 ± 1.4 | 2.6 ± 1.1 | **<0.001** |
| NSAID, daily use | 59 (51.3) | 17 (42.5) | **0.01** |
| csDMARD | 11 (9.6) | 7 (17.5) | 0.35 |
| bDMARD | 16 (13.9) | 9 (22.5) | 0.33 |
| csDMARD and/or bDMARD | 5 (4.3) | 3 (7.5) | 0.44 |
| Glucocorticosteroids | 29 (25.2) ° | 7 (17.5) | 0.31 |
| r-axSpA drug treatment^#^ | 43 (37.3) | 15 (37.5) | 0.99 |
| **Comorbidity and CV-related variables** | | | |
| SBP, mmHg | 140.0 ± 17.9 | 125.0 ± 15.8 | **<0.001** |
| DBP, mmHg | 77.0 ± 9.7 | 75.0 ± 8.0 | 0.08 |
| Hypertension | 61 (53.0) | 8 (20.0) | **<0.001** |
| Previous myocardial infarction | 6 (5.2) | 0 (0.0) | 0.14 |
| Surgical myocardial revascularisation | 5 (4.3) | 0 (0.0) | 0.18 |
| Previous stroke | 2 (1.7) | 0 (0.0) | 0.40 |
| Previous CV event^§^ | 9 (7.8) | 0 (0.0) | 0.07 |
| Diabetes mellitus | 11 (9.6) | 0 (0.0) | **0.04** |
| Dyslipidemia | 21 (18.2) | 1 (2.5) | **0.01** |
| Left cIMT, mm | 0.8 ± 0.2° | 0.6 ± 0.1 | **<0.001** |
| Right cIMT, mm | 0.8 ± 0.1 | 0.6 ± 0.1 | **<0.001** |
| Mean cIMT, mm | 0.8 ± 0.1° | 0.6 ± 0.1 | **<0.001** |
| **Markers of inflammation** | | | |
| hs-CRP, mg/L | 4.9 ± 6.6 | 3.8 ± 4.1 | 0.30 |
| ESR, mm/h | 15.1 ± 12.5 | 10.2 ± 8.6 | **0.02** |
| IL-6 pg/mL | 2.5 ± 8.6 | 2.1 ± 1.0 | 0.77 |
| WBCs, 10^9^/L | 7.1 ± 1.8 | 6.6 ± 1.6 | 0.12 |
| Monocytes, 10^9^/L | 0.6 ± 0.2 | 0.5 ± 0.1 | **0.01** |
| Lymphocytes, 10^9^/L | 1.9 ± 0.7 | 1.9 ± 0.5 | 0.88 |
| Basophils, 10^9^/L | 0.1 ± 0.0 | 0.1 ± 0.0 | 0.08 |
| Eosinophils, 10^9^/L | 0.2 ± 0.1 | 0.2 ± 0.2 | 0.68 |
| Neutrophils, 10^9^/L | 4.3 ± 1.5 | 3.9 ± 1.4 | 0.13 |

Values are mean ± SD or n (%).

r-axSpA: radiographic axial spondyloarthritis; BMI: Body Mass Index, HLA B-27 Human Leukocyte Antigen B-27, BASDAI: Bath Ankylosing Disease Activity Index; ASDAS-CRP Ankylosing Spondylitis Disease Activity Score with C-reactive protein, BASFI: Bath Ankylosing Spondylitis Functional Index; BASMI: Bath Ankylosing Spondylitis Metrology Index; NSAID: nonsteroidal anti-inflammatory drug; csDMARD: conventional synthetic disease-modifying antirheumatic drug; bDMARD: biological disease-modifying antirheumatic drugs; SBP; systolic blood pressure, DBP: Diastolic blood pressure, CV: cardiovascular, cIMT: carotid intima media thickness, hs-CRP: high sensitivity C-reactive protein; ESR: erythrocyte sedimentation rate; IL-6: interlukin-6, WBCs: white blood cells.

§ Previous CV event variable comprises of previous myocardial infarction, stroke, and surgical myocardial revasculisation

# r-axSpA drug treatment comprises treatment with DMARDs and or glucocorticosteroids

° 1 value missing

**Table 2** Sensitivity analysis; radiographic axial spondyloarthritis Backbone patient without previous cardiovascular disease (n=146)

|  |  | **Mean cIMT, mm** | |  |
| --- | --- | --- | --- | --- |
|  |  | **Regression coefficient, β unstandardized (CI 95%)** | **R^2^ adjusted** | **P value** |
| **^§^ hs-CRP,** mg/L. | **Model 1** | 0.010 (-0.041, 0.061) | 0.001^ | 0.70 |
|  | **Model 2** | -0.00069 (-0.040, 0.039) | 0.41 | 0.97 |
|  | **Model 3** | -0.012 (-0.052, 0.028) | 0.45 | 0.54 |
| **^§^ ESR,** mm/h | **Model 1** | 0.031 (-0.028, 0.089) | 0.007^ | 0.30 |
|  | **Model 2** | -0.00066 (-0.048, 0.046) | 0.41 | 0.98 |
|  | **Model 3** | 0.0038 (-0.047, 0.055) | 0.45 | 0.88 |
| **^§^ IL-6,** pg/mL | **Model 1** | 0.019 (-0.0064, 0.043) | 0.015^ | 0.14 |
|  | **Model 2** | 0.003 (-0.017, 0.022) | 0.41 | 0.77 |
|  | **Model 3** | -0.0029 (-0.024, 0.018) | 0.45 | 0.78 |
| **WBCs,** 10^9^/L | **Model 1** | 0.017 (0.0036, 0.030) | 0.043^ | **0.013** |
|  | **Model 2** | 0.017 (0.0069, 0.027) | 0.45 | **0.001** |
|  | **Model 3** | 0.018 (0.0072, 0.028) | 0.49 | **0.001** |
| **Monocytes,** 10^9^/L | **Model 1** | 0.26 (0.12, 0.40) | 0.082^ | **<0.001** |
|  | **Model 2** | 0.12 (0.0065, 0.24) | 0.43 | **0.039** |
|  | **Model 3** | 0.11 (-0.016, 0.23) | 0.46 | 0.089 |
| **ASDAS-CRP** | **Model 1** | 0.000010 (-0.035, 0.035) | 0.000^ | 1.00 |
|  | **Model 2** | -0.0039 (-0.032, 0.024) | 0.41 | 0.78 |
|  | **Model 3** | -0.016 (-0.043, 0.012) | 0.45 | 0.26 |

cIMT: carotid intima media thickness, CI 95%: 95% confidence interval, hs-CRP: high sensitivity C-reactive protein, ESR: erythrocyte sedimentation rate, IL-6: Interleukin 6, WBC:s white blood cells, BMI: body mass index, ASDAS: ankylosing spondylitis disease activity score, DMARDs: disease modifying anti-rheumatic drugs, CV: cardiovascular, MI: myocardial infarction.

^ R^2^ Values are unadjusted

* ASDAS-CRP was not used in these adjustment models due to co-linearity with hs-CRP

**^§^** Independent variables were log adjusted

Adjustment models.

1. Unadjusted
2. Age, sex, smoking status
3. Age, sex, smoking status, ASDAS-CRP, BMI, hypertension, dyslipidemia, diabetes mellitus, r-axSpA drug treatment (DMARDs and glucocorticoids)
